# Supplementary material for: Membrane interactions of the globular domain and the hypervariable region of KRAS4b define its unique diffusion behavior
Source: eLife. 2020 Jan 20;9:e47654. doi: 10.7554/eLife.47654 (PMC7060043; doi:10.7554/eLife.47654)
Supplement: Supplementary file 2. [file elife-47654-supp2.docx]

| **Key Resources Table** | | | | |
| --- | --- | --- | --- | --- |
| Reagent type (species) or resource | Designation | Source or reference | Identifiers | Additional information |
| gene (*Homo sapiens*) | *KRAS4a* |  | NM_033360.4; NP_203524.1 |  |
| gene (*Homo sapiens*) | *KRAS4b* |  | NM_004985.5; NP_004976.2 |  |
| gene (*Homo sapiens*) | *HRAS* |  | NM_005343.4; NP_005334.1 |  |
| gene (*Homo sapiens*) | *NRAS* |  | NM_002524.5; NP_002515.1 |  |
| cell line (*Homo sapiens*) | HeLa | ATCC | ATCC:CCL-2;  RRID:CVCL_0030 | Cervix, RAS WT |
| cell line (*Homo sapiens*) | Dox-inducible Halotag-Kras4b HeLa | This paper | NCI RAS Program ID: RPZ26926 | See Materials and Methods: Generation of Dox-inducible HaloTag KRAS4b HeLa Cell Pool |
| cell line (*Homo sapiens*) | SU86.86 | Cyril Benes/MGH | MGH:955;  RRID:CVCL_3881;  NCI RAS Program ID: RPZ25505 | Pancreas, KRAS G12D mutation |
| cell line (*Homo sapiens*) | HPNE | ATCC | ATCC:CRL-4036;  RRID:CVCL_C467;  NCI RAS Program ID: RPZ27105 | Pancreas, KRAS G12D mutation |
| cell line (*Homo sapiens*) | Panc-1 | ATCC | ATCC: CRL-1469;  RRID:CVCL_0480;  NCI RAS Program ID: RPZ25839 | Pancreas, KRAS G12D mutation |
| cell line (*M. musculus*) | MEF *Kras fl/fl* DU1473 | University of California San Francisco | NCI RAS Program ID: RPZ113 | Mouse embryonic fibroblast, *NRAS -/-, HRAS -/-, KRAS fl/fl* |
| cell line (*M. musculus*) | MEF Halotag-KRAS4b | This paper | NCI RAS Program ID: RPZ26147 | See Materials and Methods: Generation of HaloTagged RAS mutant MEFs |
| cell line (*M. musculus*) | MEF Halotag-KRAS4a | This paper | NCI RAS Program ID: RPZ27478 | See Materials and Methods: Generation of HaloTagged RAS mutant MEFs |
| cell line (*M. musculus*) | MEF Halotag-HRAS | This paper | NCI RAS Program ID: RPZ27482 | See Materials and Methods: Generation of HaloTagged RAS mutant MEFs |
| cell line (*M. musculus*) | MEF Halotag-NRAS | This paper | NCI RAS Program ID: RPZ27480 | See Materials and Methods: Generation of HaloTagged RAS mutant MEFs |
| antibody | p44/42 MAPK (Erk 1/2) mouse monoclonal | Cell Signaling Technology | Cell Signaling:4696;  RRID:AB_390780 | WB (1:1000) |
| antibody | Phospho-p44/42 MAPK (Erk 1/2) rabbit monoclonal | Cell Signaling Technology | Cell Signaling:4370;  RRID:AB_2315112 | WB (1:2000) |
| antibody | MEK 1/2 mouse monoclonal | Cell Signaling Technology | Cell Signaling:4694;  RRID:AB_10695868 | WB (1:1000) |
| antibody | Phospho-MEK 1/2 rabbit monoclonal | Cell Signaling Technology | Cell Signaling:9154;  RRID:AB_2138017 | WB (1:1000) |
| antibody | Akt (pan) mouse monoclonal | Cell Signaling Technology | Cell Signaling:2920;  RRID:AB_1147620 | WB (1:1000) |
| antibody | Phospho-Akt (Ser473) rabbit monoclonal | Cell Signaling Technology | Cell Signaling:4060;  RRID:AB_2315049 | WB (1:2000) |
| antibody | Anti-Ras mouse monoclonal | Thermo Fisher Scientific | Thermo Fisher Scientific:16117 | WB (1:1000)  Sourced from Active Ras Pull-Down and Detection Kit |
| antibody | Anti-HaloTag mouse monoclonal | Promega | Promega: G9211;  RRID:AB_2688011 | WB (1:1000) |
| antibody | Anti-Vinculin, mouse monoclonal | Sigma-Aldrich | Sigma-Aldrich:V9131  RRID:AB_477629 | WB (1:1000) |
| antibody | Goat anti-mouse 680RD | LI-COR | LI-COR:926-68070;  RRID:AB_10956588 | WB (1:10,000) |
| antibody | Goat anti-rabbit 800CW | LI-COR | LI-COR: 926-32211;  RRID:AB_621843 | WB (1:10,000) |
| recombinant DNA reagent | HaloTag-Hs.KRAS4b (1-188) | This paper | Clone ID: R733-M75-303 | See Materials and Methods: Protein Constructs |
| recombinant DNA reagent | HaloTag-Hs.KRAS4b (1-188) G12D | This paper | Clone ID: R703-M74-303 | See Materials and Methods: Protein Constructs |
| recombinant DNA reagent | HaloTag-Hs.KRAS4b (1-188) Q61R | This paper | Clone ID: R703-M78-303 | See Materials and Methods: Protein Constructs |
| recombinant DNA reagent | HaloTag-Hs.KRAS4b (1-188) Y40C | This paper | Clone ID: R713-M73-303 | See Materials and Methods: Protein Constructs |
| recombinant DNA reagent | HaloTag-Hs.KRAS4b (1-188) C185S | This paper | Clone ID: R750-M65-303 | See Materials and Methods: Protein Constructs |
| recombinant DNA reagent | HaloTag-Hs.KRAS4b (1-188) Y40C/Q61R | This paper | Clone ID: R743-M53-303 | See Materials and Methods: Protein Constructs |
| recombinant DNA reagent | HaloTag-Hs.KRAS4a (1-189) | This paper | Clone ID: R713-M85-303 | See Materials and Methods: Protein Constructs |
| recombinant DNA reagent | HaloTag-Hs.HRAS (1-189)  (plasmid) | This paper | Clone ID: R713-M88-303 | See Materials and Methods: Protein Constructs |
| recombinant DNA reagent | HaloTag-Hs.NRAS (1-189)  (plasmid) | This paper | Clone ID: R713-M91-303 | See Materials and Methods: Protein Constructs |
| recombinant DNA reagent | HaloTag-Hs.KRAS4b (167-188)  (plasmid) | This paper | Clone ID: R703-M80-303 | See Materials and Methods: Protein Constructs |
| recombinant DNA reagent | HaloTag-Hs.KRAS4a (167-189)  (plasmid) | This paper | Clone ID: R713-M48-303 | See Materials and Methods: Protein Constructs |
| recombinant DNA reagent | HaloTag-Hs.HRAS (167-189)  (plasmid) | This paper | Clone ID: R713-M50-303 | See Materials and Methods: Protein Constructs |
| recombinant DNA reagent | HaloTag-Hs.NRAS (167-189)  (plasmid) | This paper | Clone ID: R713-M52-303 | See Materials and Methods: Protein Constructs |
| recombinant DNA reagent | HaloTag-Hs.KRAS4b (167-188) 176A/K178A/K180A (3A)  (plasmid) | This paper | Clone ID: R733-M31-303 | See Materials and Methods: Protein Constructs |
| recombinant DNA reagent | HaloTag-Hs.KRAS4b (167-188) K175E/K176E/K177E (3Ea)  (plasmid) | This paper | Clone ID: R733-M65-303 | See Materials and Methods: Protein Constructs |
| recombinant DNA reagent | HaloTag-Hs.KRAS4b (167-188) K178E/K179E/K180E (3Eb)  (plasmid) | This paper | Clone ID: R733-M67-303 | See Materials and Methods: Protein Constructs |
| recombinant DNA reagent | HaloTag-Hs.KRAS4b (167-188) K169A/K172A/K176A/K178A/K180A (5A)  (plasmid) | This paper | Clone ID: R733-M33-303 | See Materials and Methods: Protein Constructs |
| recombinant DNA reagent | HaloTag-Hs.KRAS4b (167-188) K169E/K172E/K176E/K178E/K180E (5Ea)  (plasmid) | This paper | Clone ID: R733-M35-303 | See Materials and Methods: Protein Constructs |
| recombinant DNA reagent | HaloTag-Hs.KRAS4b (167-188) K176E/K178E/K180E/K182E/K184E (5Eb)  (plasmid) | This paper | Clone ID: R733-M37-303 | See Materials and Methods: Protein Constructs |
| recombinant DNA reagent | HaloTag-Hs.KRAS4b (1-188) D126E/T127S/K128R  (plasmid) | This paper | Clone ID: R743-M56-303 | See Materials and Methods: Protein Constructs |
| recombinant DNA reagent | HaloTag-Hs.KRAS4b (1-188) E91G/H94N/H95K  (plasmid) | This paper | Clone ID: R743-M55-303 | See Materials and Methods: Protein Constructs |
| recombinant DNA reagent | HaloTag-Hs.KRAS4b (1-188) Q131H/D132E/R135K  (plasmid) | This paper | Clone ID: R743-M57-303 | See Materials and Methods: Protein Constructs |
| recombinant DNA reagent | HaloTag-Hs.KRAS4b (1-188) TetON promoter  (plasmid) | This paper | Clone ID: R713-M15-663 | See Materials and Methods: Protein Constructs; Generation of Dox-inducible HeLas |
| recombinant DNA reagent | CMV TetON3G promoter  (plasmid) | This paper | Clone ID: R980-M38-658 | See Materials and Methods: Protein Constructs; Generation of Dox-inducible HeLas |
| software, algorithm | ImageJ | ImageJ (<https://imagej.nih.gov/ij/>) | RRID:SCR_003070 |  |
| software, algorithm | TrackMate | ImageJ  (<https://imagej.net/TrackMate>) |  | ImageJ Plugin |
| software, algorithm | Localizer | (Dedecker et. al, 2012)  (<https://doi.org/10.1117/1.JBO.17.12.126008>) |  | ImageJ Plugin |
| software, algorithm | vbSPT | vbSPT (<http://vbspt.sourceforge.net/>) | RRID:SCR_017554 |  |
| software, algorithm | Matlab | Matlab  (<https://www.mathworks.com/products/matlab.html>) | RRID:SCR_001622 |  |
| software, algorithm | TrackArt | (Matysik and Kraut, 2014)  (<https://doi.org/10.1186/1756-0500-7-274>) |  |  |
| software, algorithm | MOE (Molecular Operating Environment) Programs | Chemical Computing Group, Inc.  (<https://www.chemcomp.com/Products.htm>) |  |  |
| software, algorithm | CHARMM36 Force Field (FF) | CHARMM (<https://www.charmm.org/charmm/>) | RRID:SCR_014892 |  |
| software, algorithm | GROMACS | GROMACS  (<http://www.gromacs.org/>) | RRID:SCR_014565 |  |
| software, algorithm | AMBER16 | Amber (<http://ambermd.org/>) | RRID:SCR_014230 |  |
| Other | JF646 dye | Promega | Promega: GA1120 | Covalent, fluorescent ligand to HaloTag (25pM) |
| Other | JF549 dye | Promega | Promega: GA1110 | Covalent, fluorescent ligand to HaloTag (25pM) |
| Other | FuGENE 6 | Promega | Promega: E2692 | Transfection Reagent |
